# Mental Health and the Association between Asthma and E-cigarette Use among Young Adults in The United States: A Mediation Analysis

**DOI:** 10.3390/ijerph17238799

**Published:** 2020-11-26

**Authors:** Abdullah M. M. Alanazi, Mohammed M. Alqahtani, Gregory Pavela, Eric W. Ford, Adam M. Leventhal, Peter S. Hendricks

**Affiliations:** 1 Rehabilitation Science, School of Health Professions, University of Alabama at Birmingham, Birmingham, AL 35294, USA; qahtani4@uab.edu; 2 Department of Respiratory Therapy, College of Applied Medical Sciences, King Saud bin Abdulaziz University for Health Sciences, Riyadh 14611, Saudi Arabia; 3 Department of Health Behavior, School of Public Health, University of Alabama at Birmingham, Birmingham, AL 35294, USA; pavela@uab.edu (G.P.); phendricks@uab.edu (P.S.H.); 4 Department of Health Care Organization and Policy, School of Public Health, University of Alabama at Birmingham, Birmingham, AL 35294, USA; ewford@uab.edu; 5 Department of Preventive Medicine, University of Southern California, Los Angeles, CA 90089, USA; adam.leventhal@usc.edu; 6 Department of Psychology, University of Southern California, Los Angeles, CA 90089, USA

**Keywords:** asthma, mental health, e-cigarettes, addiction, substance use, mediation

## Abstract

Background: Asthma is associated with a greater likelihood of e-cigarette use among young adults, which may increase the risk of pulmonary complications. Because substance use trajectories emerge in early adulthood, it is important to identify factors that may be important in addressing this new public health threat. One such factor may be poor mental health. Methods: Data were extracted from the 2018 Behavioral Risk Factor Surveillance System (BRFSS). Current and former asthma status was measured by self-reported lifetime and current asthma status; mental health functioning was measured by the number of self-reported bad mental health days during the past 30 days; e-cigarette use was measured by self-reported current e-cigarette use. We tested the hypothesis that mental health mediates the association between asthma status and e-cigarette use among young adults using structural equation modeling. Results: The prevalence of e-cigarette use was significantly higher among young adults with current (9.90%) or former asthma (13.09%) than those without asthma (9.58%). Furthermore, the number of bad mental health days in the past 30 days was significantly greater among young adults with current or former asthma than among those without asthma (Mean (Standard Deviation): 6.85 (0.42), 4.18 (0.85) versus 3.83 (0.17)), respectively. Finally, we found a statistically significant indirect effect of asthma on the likelihood of e-cigarette use through mental health such that the higher prevalence of e-cigarette use among those with current or former asthma was statistically accounted for by a greater number of bad mental health days in the past 30 days. Conclusions: Consistent with mediation, poorer mental health accounted for the higher prevalence of e-cigarette use among those with asthma. However, longitudinal studies are needed to interrogate causal relationships, including the effects of e-cigarette use on mental health. Mental health services may play an important role in improving health and wellbeing in this vulnerable population.

## 1. Introduction

Asthma is a chronic pulmonary disease that exacerbates airway hypersensitivity and significantly increases the risk of pulmonary complications, the severity and onset of which are adversely affected by a number of risk behaviors [1,2,3,4]. In particular, combustible tobacco products may aggravate asthma symptoms and accelerate adult-onset asthma [5,6,7,8]. Although combustible cigarette use has declined in recent years [9,10], the prevalence of e-cigarette use has risen dramatically in the United States [11], especially among middle and high school aged students [9,10,12]. The rapid increase in e-cigarette use has prompted concern about potential adverse health effects, particularly among young adults already at risk for pulmonary complications [13,14,15,16,17,18,19].

The emphasis on e-cigarette use among young adults is vital, as substance use trajectories often emerge in young adulthood [20,21]. Indeed, the U.S. Surgeon General specifically acknowledged e-cigarette use among youth as a public health epidemic in 2018 [22]. Jones et al. (2019) reported that from 2003 to 2017, the smoking rate among youth with asthma decreased from 25.2% to 10.7%, whereas the rate of e-cigarette use increased from 11.7% to 27.5% [23]. These findings suggest that the use of e-cigarettes among young adults with asthma may be particularly problematic.

E-cigarette exposure can trigger pathologic responses in the lung, including airway irritation, increased mucus secretion, and upregulation of pro-inflammatory mediators [24]. One clinical study indicated that e-cigarette and combustible cigarette use trigger the nicotine-dependent release of proteases from pulmonary immune cells and increase proteolysis in a similar fashion [25]. Of particular relevance to the present study, epidemiologic evidence suggests a link between e-cigarette use and asthma. Recent analyses of data from large national databases found that e-cigarette use is associated with increased risk of asthma or other respiratory disease (COPD, chronic bronchitis, or emphysema) among adults in the United States [26,27]. Indeed, the likelihood of reporting asthma was higher among daily e-cigarette users than among never users (odds ratio = 1.82, 95% confidence interval (CI) (1.23, 2.66)) [14]. Similarly, another recent study found that current e-cigarette use was associated with both current asthma and ever having asthma among adolescents [16]. Recent case reports of severe lung disease secondary to vaping in both seemingly healthy individuals [28] and those with asthma [29] further highlights the need for concern regarding the adverse pulmonary effects of e-cigarette use.

The prevention of e-cigarette use and associated adverse effects among young adults with asthma requires the identification of motivating factors for e-cigarette use in this population, as a better understanding as to why individuals with asthma are more likely to use e-cigarettes will allow for more targeted interventions. A leading theory of addiction motivation posits that individuals use addictive substances, including nicotine, to escape or avoid negative feelings [30]. As a wide spectrum of mental health conditions have been associated with asthma, including depression [31,32], anxiety [31,32,33,34], and mood disorders [34,35], individuals with asthma may use e-cigarettes as a form of self-medication. If true, this suggests educational initiatives and enhanced mental health services as possible measures to both improve wellbeing and promote respiratory health via the prevention of e-cigarette use.

The purpose of this study was to test the hypothesis that mental health functioning would account for the relationship between asthma status and e-cigarette use among those aged 18 to 24 years old. Specifically, we hypothesized that the association between asthma status (both current and former) and e-cigarette use is mediated by poorer mental health functioning. A cross-sectional, nationally representative study of U.S. adults was analyzed using structural equation modelling.

## 2. Materials and Methods

### 2.1. Data Sources

We analyzed 2018 data from a national database from the Center for Disease Control and Prevention (CDC) in the United States: The Behavioral Risk Factor Surveillance System (BRFSS). The BRFSS collects self-reported cross-sectional data through a telephone survey of adults (≥18 years of age) across all 50 states and includes questions that assess the prevalence of chronic health conditions, including asthma, and health-related risk behaviors [36]. Our analytic sample was limited to respondents between the ages of 18–24 years old in order to test our hypotheses in young adults.

### 2.2. Measures

#### 2.2.1. Asthma Status

Asthma status was determined by asking participants: “Has a doctor, nurse, or other health professional EVER told you that you had any of the following?” Using the question assessing asthma status, “Ever told you had asthma?”, an indicator of lifetime asthma status was created. Then, to determine current asthma status, those who answered “yes” to the lifetime asthma question were asked, “Do you still have asthma?” Based on the pattern of answers to the above questions, a trichotomous indicator of asthma status was created as follows; no asthma (lifetime asthma = 0); former asthma (lifetime asthma = 1, current asthma = 0); and current asthma (lifetime asthma = 1, current asthma = 1). Those who answered “Not sure” were treated as missing.

#### 2.2.2. E-Cigarette Use

To assess e-cigarette use, participants were asked: “Have you ever used an e-cigarette or other electronic vaping product, even just one time, in your entire life?” Those who answered “yes” were then asked, “Do you now use e-cigarettes every day, some days, or not at all?” A dichotomous indicator of e-cigarette use was created as follows; no current e-cigarette use (“lifetime e-cigarette use” = 0 or “lifetime e-cigarette use” = 1 and both “current every day use” = 0 and “current someday e-cigarette use” = 0) or current e-cigarette use (“lifetime e-cigarette use” = 1 and either “current every day use” = 1 or “someday e-cigarette use” = 1). We used current e-cigarette use, not lifetime e-cigarette use, as the dependent variable because the mediator of interest, mental health functioning, was exclusive to the past 30 days, complicating any time-ordering assumptions between lifetime e-cigarette use and mental health. Although the survey specified that this question concerned electronic vaping products for nicotine use only, and not cannabis use [36], the survey did not ask participants to specify which type of electronic device(s) they used. Therefore, we could not differentiate between specific devices in our analyses.

#### 2.2.3. Mental Health Functioning

Mental health functioning was determined by asking “Now thinking about your mental health, which includes stress, depression, and problems with emotions, for how many days during the past 30 days was your mental health not good?” Answers ranged from 0–30 days, and this variable was analyzed as a continuous variable.

#### 2.2.4. Covariates

Analyses adjusted for sex (male or female), educational attainment (did not graduate high school, graduated high school, attended college or technical school, or graduated from college or technical school), and race (White, Black, American Indian/Alaskan native, Asian, or Other). Analyses additionally controlled for cigarette smoking status (0 = never (reference category), 1 = former smoker, 2 = current someday smoker, and 3 = current every day smoker), and other chronic health conditions ((COPD, diabetes, kidney disease, arthritis, skin cancer and other cancers); each chronic health condition was coded as 0 = no (reference category), and 1 = yes) as obtained from the BRFSS survey.

### 2.3. Statistical Analysis

We used Stata version 16.1 to conduct descriptive statistics, one-way analysis of variance (ANOVA), and chi-square tests of independence. Prevalence is reported in weighted percentages and the reported N is adjusted using the weighted analysis to account for the complex survey design. We used structural equation modeling to test the indirect effects of asthma status (currently had asthma and formerly had asthma vs. never had asthma) on current e-cigarette use through mental health functioning. The model was weighted to account for the complex survey design of BRFFS and statistical inferences about the indirect effects were based on the Sobel test [37,38]. The analyses used the subpopulation command in STATA to construct estimates for the young adult sample using the age category of 18 to 24 years old only. Missing values were handled via case-wise deletion and *p*-values < 0.05 were considered statistically significant.

## 3. Results

### 3.1. Sample Characteristics

Sociodemographic characteristics of respondents by asthma status are shown in Table 1. As noted in Table 1, those with current or former asthma were more likely to report current e-cigarette use and reported a greater number of bad mental health days in the past 30 days relative to the never asthma group. Those with current asthma were more likely to be female, Black, and had lower educational attainment than those with former asthma and those who never had asthma. Those with former asthma and never asthma were mostly male and White. Finally, as compared to the never asthma group, the former asthma group was more likely to report current smoking, and both the former and current asthma groups were more likely to report other chronic health conditions, with the prevalence of other chronic health conditions being greatest among the current asthma group. 

Across asthma statuses, the weighted percentages of missing data for e-cigarette use (range = 3.07% to 6.31%), bad mental health days in the past 30 days (range = 1.47% to 1.80%), sex (range = 0.03% to 0.09%), educational attainment (range = 0.14% to 0.30%), race (range = 2.27% to 4.50%), smoking status (range = 2.74% to 5.59%), COPD (range = 0.32% to 0.80%), diabetes (range = 0.12% to 0.26%), chronic kidney disease (range = 0.07% to 0.55%), arthritis (range = 0.30% to 0.73%), skin cancer (range = 0.08% to 0.17%), and other cancers (range = 0.08% to 0.17%) were low and did not significantly differ.

Table 2 shows results of weighted multivariate models predicting e-cigarette use from asthma status, mental health functioning, and other covariates. Asthma status was not independently associated with e-cigarette use. However, poorer mental health functioning and former smoker status, current someday smoker status, and current every day smoker status were each associated with an increased likelihood of e-cigarette use. No other variables were associated with e-cigarette use.

### 3.2. Indirect Effects

**Figure 1:**
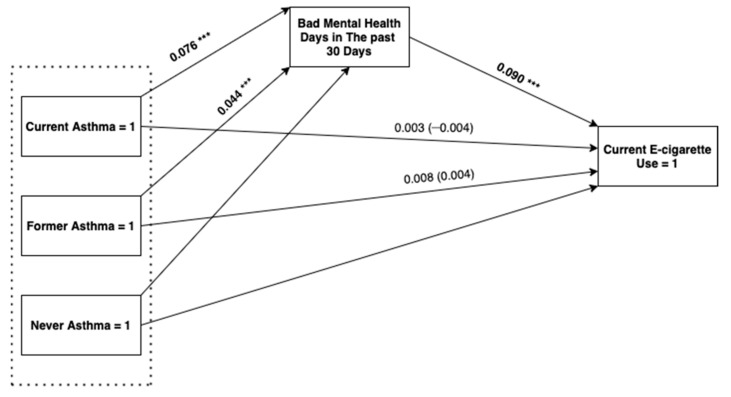
Structural equation model testing the indirect effects of current asthma status (0 = never, 1 = current) and former asthma status (0 = never, 1 = former) on current e-cigarette use (0 = no, and 1 = yes) through bad mental health days in the past 30 days among young adults. The model controlled for sociodemographic variables (sex, educational attainment, and race), smoking status, and other chronic health conditions (COPD, diabetes, chronic kidney disease, arthritis, skin cancer, and other cancers). Reported statistics are standardized regression coefficients. The values in parentheses are the direct effects of current/former asthma. Weighted N = 27,762,042. *** *p* < 0.001.

Figure 1 reports the results of the analysis testing whether mental health functioning accounted for the association between asthma status and current e-cigarette use. Because asthma status is a multicategory antecedent variable, indirect and direct effects are relative to the effect of the excluded reference category, which in this case was never having asthma [38].

The direct effect of current asthma on odds of current e-cigarette use was not statistically significant (standardized beta = −0.004, standard error (SE) = 0.017, 95% CI (−0.037, 0.029)); however, current asthma was associated with a greater number of bad mental health days in the past 30 days relative to those who never had asthma (standardized beta = 0.076, SE = 0.014, 95% CI (0.049, 0.103)) which was, in turn, a significant predictor of current e-cigarette use. Consistent with mediation, the indirect effect of current asthma on the likelihood of e-cigarette use through mental health functioning was significant (standardized beta = 0.007, SE = 0.002, 95% CI (0.003, 0.011)).

As with current asthma status, the direct effect of former asthma status on odds of current e-cigarette use was not statistically significant (standardized beta = 0.004, SE = 0.017, 95% CI (−0.029, 0.038]). Formerly having asthma was, however, associated with a greater number of bad mental health days (standardized beta = 0.044, SE = 0.011, 95% CI (0.022, 0.065)). Consistent with mediation, the indirect effect of former asthma through mental health functioning was significant (standardized beta = 0.004, standard error = 0.001, 95% CI (0.001, 0.007)).

## 4. Discussion

In this nationally representative sample of young adults in the United States, we found that poorer mental health functioning accounted for the association between asthma and e-cigarette use. In 2019, the CDC documented 52 deaths from severe lung injury associated with the use of e-cigarettes or vaping products [39], prompting the release of an interim guidance report [40]. Although most of the severe lung injuries were linked to vaping cannabis solutions containing vitamin E acetate [41], these cases brought attention to the growing body of evidence supporting the pathologic effects of e-cigarettes in general, especially in the lung [27,28], and the need to prevent this behavior in vulnerable populations.

The evidence supporting a link between mental health functioning and the use of addictive substances, including nicotine [42,43], prompted us to test whether mental health functioning is associated with e-cigarette use in young adults with asthma. Our findings, therefore, suggest that among young adults with asthma, poorer mental health functioning may contribute to the increased use of e-cigarettes. Consistent with a leading contemporary theory of addiction motivation, the escape of avoidance of negative affect may form the motivational basis for e-cigarette use [30].

However, it is noted that because the data were collected cross-sectionally, we cannot rule out reverse causal effects. In particular, specific irritants in e-cigarette vapor may cause inflammation in the hypersensitive airways of individuals with asthma that, in turn, may increase the number of bad mental health days. Further, e-cigarette use could potentially cause poor mental health considering the known effects of nicotine on the developing brain’s emotional and cognitive processing circuits. [44,45]. Consistent with this view, a recent longitudinal analysis found that whereas psychological well-being is associated with a reduced likelihood of smoking, smoking is associated with lower psychological well-being [46]. It is likely, therefore, that poor mental health is both a cause and consequence of e-cigarette use. In the current study, the dichotomous indicator of e-cigarette use was not tested as a mediator of the relationship between asthmas status and mental health functioning as analyses involving dichotomous mediators are not yet fully developed or widely implemented in statistical analysis programs. It should also be noted that the reported effects were modest in size. However, small effects can have meaningful impacts at the population level [47]. Indeed, considering the rising prevalence of e-cigarette use among young adults with asthma, even modest effects could account for e-cigarette use among thousands of these vulnerable individuals.

Our study has several limiting factors. The data analyzed in this study were generated from a cross-sectional population survey, limiting our ability to infer causality, and the responses were self-reported, which increases the risk of social desirability and other biases [47]. Furthermore, asthma diagnosis was based on a subjective measure, not a clinical diagnosis that also indicates the degree of severity, acuity and treatment status. Finally, the data could not specify the type of mental health problems since the measure was broad to stress, depression, and problems with emotions.

## 5. Conclusions

In conclusion, our results offer a potential understanding of the mechanisms underlying the relationship between asthma and the use of e-cigarettes. Specifically, we identified mental health functioning as a potential mediator of the relationship between current/former asthma and current use of e-cigarettes among young adults. This research is a crucial first step in identifying potential interventions to reduce e-cigarette use in this special population and suggests that the provision and utilization of mental health services among individuals with asthma may decrease their risk of using e-cigarettes. However, longitudinal studies with validated measurements are needed to more definitively establish the causal relationships between asthma, mental health functioning, and e-cigarette use.

## Figures and Tables

**Table 1:** Sociodemographic characteristics of the population of young adults (18 to 24 years old) by asthma status.

Variable	Asthma = Never, Weighted %/Mean (SD)	Asthma = Former, Weighted %/Mean (SD)	Asthma = Current, Weighted %/Mean (SD)	*F* Ratio/*t*-Value, *p*-Value
E-cigarette use				419.8, 0.015
No current use	90.42	86.91	90.10	
Current use	9.58	13.09	9.90	
Bad mental health days in the past 30 days	3.83 (0.17)	4.18 (0.85)	6.85 (0.42)	56.41, <0.001
Sex				6162.40, <0.001
Male	51.86	64.40	37.55	
Female	48.14	35.60	62.45	
Educational attainment				16.07.1, <0.001
Did not graduate high school	10.90	9.11	15.27	
Graduated high school	38.15	40.46	35.96	
Attended college or technical school	39.04	39.53	38.37	
Graduated from college or technical school	11.91	10.90	10.40	
Race				2052.1, <0.001
White	67.58	69.79	69.12	
Black	14.51	13.78	18.78	
Asian	10.17	9.55	5.01	
American Indian/ Alaskan Native	1.89	1.68	2.54	
Other	5.85	5.20	4.55	
Smoking status				1488.0, <0.001
Never smoker	81.75	76.96	81.08	
Former smoker	6.37	6.11	6.36	
Current someday smoker	6.65	9.84	7.12	
Current every day smoker	5.22	7.09	5.45	
COPD				5841.18, <0.001
No	98.91	96.78	93.88	
Yes	1.09	3.22	6.12	
Diabetes				215.44, 0.090
No	99.01	98.54	98.30	
Yes	0.99	1.46	1.70	
Chronic kidney disease				777.10, 0.012
No	99.25	98.10	98.20	
Yes	0.75	1.90	1.80	
Arthritis				4622.37, <0.001
No	97.73	95.68	91.82	
Yes	2.27	4.32	8.18	
Skin cancer				1412.07, <0.001
No	99.73	99.40	98.48	
Yes	0.27	0.59	1.52	
Other cancers				580.96, <0.001
No	99.51	99.39	98.54	
Yes	0.49	0.61	1.46	
	_Weighted_N= 26,017,419	_Weighted_N = 2,503,503	_Weighted_N = 3,200,681	

e-cigarette, electronic cigarette; COPD, chronic obstructive pulmonary disease; SD, standard deviation. Weighted N = 31,721,603.

**Table 2:** Weighted multivariate models of adjusted odds of current e-cigarette use among young adults predicted by asthma status, mental health functioning, and other covariates.

Variable	Current E-Cigarette Use	
	aOR	95%CI
Current asthma	1.001	0.784, 1.278
Former asthma	1.069	0.786, 1.455
Bad mental health days in the past 30 days	1.020	1.011, 1.028
Smoking status		
Never smoker	Ref	Ref
Former smoker	5.053	3.995, 6.393
Current someday smoker	3.996	3.176, 5.027
Current every day smoker	5.465	4.219, 7.079

Other covariates: sex, educational attainment, race, COPD, diabetes, chronic kidney disease, arthritis, skin cancer, and other cancers. E-cigarette, electronic cigarette; aOR, adjusted odd ratio; 95% CI, 95% confidence interval; Ref, reference.
